# Do Restorative Strategies and Delayed Restoration Improve the Bond Strength to Biodentine? In Vitro Study on Adhesive Restorations on a Calcium Silicate-Based Cement

**DOI:** 10.3290/j.jad.c_2323

**Published:** 2025-11-18

**Authors:** Eugenia Baena, Nuria Escribano, Victoria Fuentes, Laura Ceballos

**Affiliations:** a Eugenia Baena Associate Professor, Health Sciences Faculty, IDIBO Research Group. Universidad Rey Juan Carlos, Alcorcón, Madrid, Spain. Conceptualization, methodology, investigation, resources, writing – original draft, writing – review and editing, supervision, project administration.; b Nuria Escribano Associate Professor, Health Sciences Faculty, IDIBO Research Group. Universidad Rey Juan Carlos, Alcorcón, Madrid, Spain. Methodology, investigation, resources, writing – original draft, writing – review and editing, visualization, project administration.; c Victoria Fuentes Associate Professor, Health Sciences Faculty, IDIBO Research Group. Universidad Rey Juan Carlos, Alcorcón, Madrid, Spain. Methodology, resources, formal analysis, data curation, writing – review and editing.; d Laura Ceballos Professor, Health Sciences Faculty, IDIBO Research Group. Universidad Rey Juan Carlos, Alcorcón, Madrid, Spain. Conceptualization, methodology, writing – original draft, writing – review and editing, supervision.

**Keywords:** Biodentine, calcium-silicate-based cement, pulpotomy, shear bond strength, vital pulp therapy

## Abstract

**Purpose**: The popularity of Biodentine (Septodont) use for vital pulp therapies requires verifying its bonding ability to other restorative materials. The null hypotheses were that different restorative materials and adhesive strategies did not influence shear bond strength (SBS) to Biodentine, nor its setting time.

**Materials and Methods:**

204 plastic molds were filled with Biodentine, half set for 12 min, and the rest for 7 days. Specimens were divided into six groups according to the strategy and material used to restore over Biodentine (n = 17): (1) SE: Universal adhesive application in self-etch mode; (2) ER: Universal adhesive in etch-and-rinse mode; (3) Bur: Roughening with a bur followed by SE; (4) AO: Airborne-particle abrading with Al_2_O_3_ particles before SE; (5) RMGIC: Restoration with a resin-modified glass-ionomer; (6) SARC: Restoration with a self-adhesive resin cement. Groups 1 to 4 were restored with a flowable bulk-fill composite. Specimens were subjected to SBS, and the mode of failure was determined. Five additional specimens were evaluated under SEM-EDX. Data were analyzed with Kruskal–Wallis and U-Mann–Whitney tests (P <0.05).

**Results:**

AO and SARC groups showed 100% pretest failures. At 12 min setting, the RMGIC group and Bonferroni correction achieved the lowest SBS values. At a 7-day setting, the Bur group registered the lowest SBS, and the groups restored with universal adhesive achieved the highest values. SBS results for the Bur and RMGIC groups were influenced by the setting time.

**Conclusion:**

Bur roughening or alumina airborne-particle abrading did not improve Biodentine adhesion to composite resin restorations, whereas the application of a universal adhesive achieved the highest SBS results regardless of the evaluated setting time.

Calcium silicate-based cements (CSCs) have become the materials of choice for vital pulp therapy (VPT), due to their antibacterial properties, ability to stimulate the pulp to form hydroxyapatite, and superior clinical outcomes compared with calcium hydroxide treatment.^12,35,38,62^ CSCs are mainly composed of calcium silicates, and a vehicle which triggers a hydration reaction allowing them to set in a humid atmosphere.^15,16^

Biodentine (Septodont, Saint-Maur-des-fossés Cedex, France), developed as a dentin replacement material, is widely used for VPT^13,16,17,27,30,47^ physical properties similar to those of dentin.^14,35,57^ Biodentine has superior compressive strength than other CSCs due to its low water/cement ratio, shorter setting time (10–12 min according to the manufacturer), higher microhardness, lower risk of tooth discoloration, and low fluid uptake and sorption.^1,24,31^ It is highly biocompatible and induces TGF-β1 secretion from dental pulp cells, related to odontoblast differentiation and reparative dentin formation.^38,39^ These are attractive features for clinicians, being considered an alternative to mineral trioxide aggregate (MTA), specifically for pulpotomies, with similar or even superior clinical outcomes.^9,25^

As with other CSCs, Biodentine requires an overlaid restoration to provide long-term mechanical strength, wear resistance, and improved esthetics.^28^ Several restorative techniques have been tested over CSCs, but a successful protocol has not yet been established.^23,40,42,51,56^ Bonding to Biodentine is a challenge due to poor mechanical interaction between direct restorative materials and Biodentine^21^ and weak or no chemical interaction with adhesive systems.^19^

The use of a glass ionomer (GI) liner over Biodentine to provide peripheral dentin sealing is a common procedure,^11,18,19,43,51^ resembling its use for the indirect pulp capping technique. Nevertheless, the benefit of this combination for the long-term performance of composite restorations is controversial according to previous clinical reports.^49^

Bonding composite resins to CSCs, mainly MTA, has been widely studied,^29,40,61^ using etch-and-rinse and self-etch adhesives. A self-etch technique has been recommended to restore over premixed MTA,^40^ due to a potential weakening effect of 37% phosphoric acid on CSC. Other studies have suggested that acid etching has no influence^33^ on MTA or may even have beneficial effects for bonding.^8,32^. Bonding over Biodentine also shows a great variability of results,^5,29,37^ therefore clinicians face the same uncertainty as with MTA. Etch-and-rinse,^19,43^ self-etch,^18,43^ and universal adhesives^19,68^ strategies have been tested. Self-adhesive flowable,^56^ bulk-fill,^19,23,56^ and nano-hybrid composites^18^ have also been used without a consensus about which procedure may be most suitable for Biodentine.^36^

Another important issue is the appropriate time to restore over Biodentine. According to the manufacturer, the recommended restoration time after Biodentine placement ranges between 7 days and 6 months to ensure the complete maturity of the CSC.^44^ A proper and immediate coronal sealing is key to the long-term success of VPTs, specifically for pulpotomy.^7,64^ The quality and durability of the bond between CSCs and the restorative materials are of clinical significance in terms of the longevity and predictability of the final restoration, and as a consequence, of the survival and healing of the dental pulp.^28,65^ Teeth should be restored in the same appointment, reducing costs, chair time, and patient discomfort.^6,18,53^

Given the lack of consensus, the aim of this *in vitro* study was to evaluate the shear bond strength (SBS) of the interface between Biodentine and several restorative materials using different adhesive strategies at two setting times: immediately (12 min) and delayed (7 days) after Biodentine placement.

The null hypotheses to be tested were (1) different adhesive strategies and restorative materials do not influence the SBS to Biodentine; and (2) the setting time has no influence on SBS.

## MATERIALS AND METHODS

**Fig 1:**
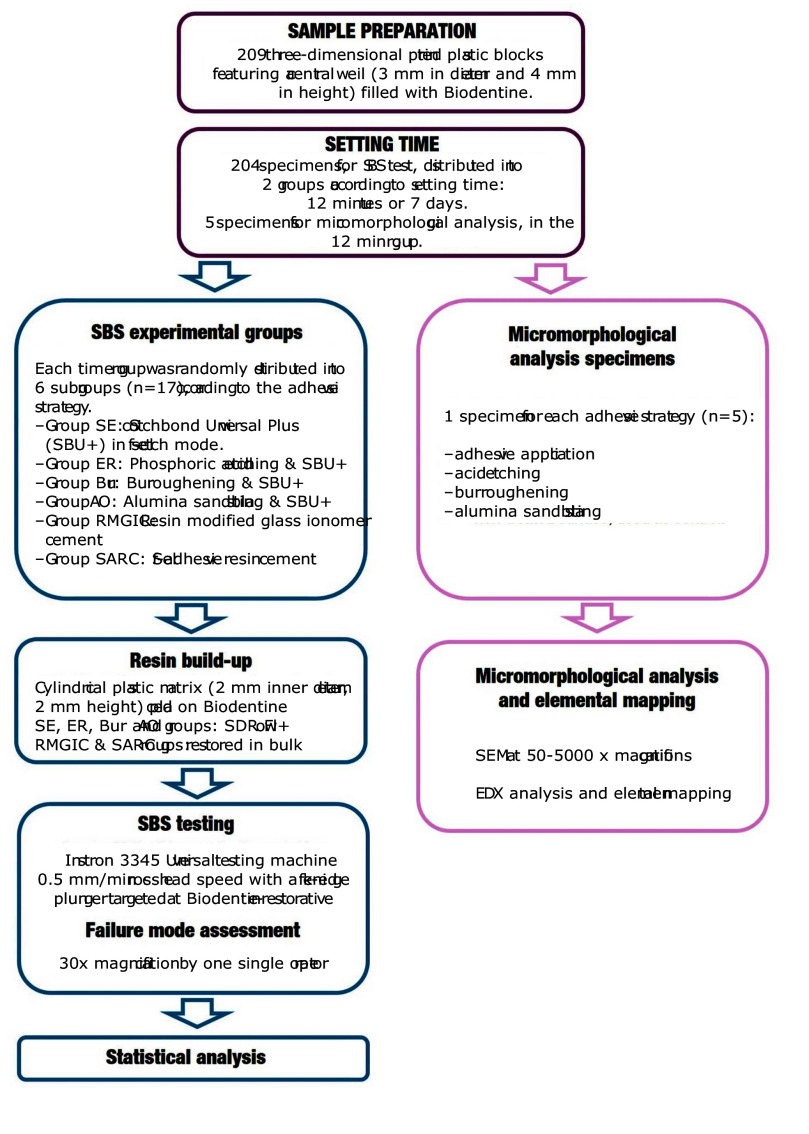
Schematic representation of the experimental workflow. The process included sample preparation using 3D-printed plastic blocks filled with Biodentine, followed by two different setting times (12 min and 7 days). Various adhesive strategies were applied prior to composite resin build-up. Specimens were then subjected to shear bond strength testing. Finally, failure modes were analyzed using microscopic evaluation. Additional specimens were allocated for micromorphological analysis of the Biodentine surface following the different adhesive strategies.

The manuscript of this laboratory study has been written according to preferred reporting items for laboratory studies in Endodontology (PRILE) 2021 guidelines.^45^ Figure 1 depicts the experimental workflow outlined in the present section.

### Specimen Preparation

The materials used in this study are listed in Table 1. A total of 204 plastic blocks with a central well (3 mm diameter, 4 mm height) were fully filled with Biodentine.

**Table 1:** Materials used in the study, along with composition and steps of application as declared by the manufacturer

Materials	Composition	Steps of application
Biodentine (Septodont, Saint-Maur-des-fossés Cedex, France)	Powder: Tricalcium and dicalcium silicates, calcium carbonate and oxide, iron oxide and zirconium oxide Liquid: Calcium chloride and hydrosoluble polymer	Pour 5 drops of single-dose container into powder capsuleMix capsule for 30 s on a mixing device (4000–4200 rotations/min)
Scotchbond Universal Etchant (Solventum, Maplewood, MN, USA)	32% phosphoric acid, fumed silica, polyethylene glycol, aluminum oxide	Apply for 15 s and water rinse for 30 sBlot-dry
Scotchbond Universal Plus (Solventum)	MDP phosphate monomer, HEMA, synthetic amorphous, silica, ethanol, water, copolymer of acrylic and itaconic acids, camphorquinone, aromatic amine, mixture of silanes, BPA derivative-free dimethacrylate monomers and copper salt	Apply and rub for 20 sGentle air stream for 5 slight-cure for 20 s
Rondoflex Sandblasting (Rondoflex, KaVo Kerr, Biberach, Germany)	Aluminum oxide particles (27 µm average particle size)	Sandblast at 10 mm distance, with 2 bar pressure
Vitrebond Plus (Solventum)	Paste: HEMA, Bis-GMA, water, initiators, radiopaque fluoroaluminosilicate glassLiquid: Resin-modified polyalkenoic acid, HEMA, water, initiators (including camphorquinone)	Dispense desired amount and mix for 15 sApply on surfacelight-cure for 20 s
RelyX Unicem 2 Automix (Solventum)	Methacrylate monomers containing phosphoric acid groups, alkaline (basic) fillers, silanated fillers (12.5 µm average particle size)	Dispense desired amountApply on surfacelight-cure for 20 s
SDR Flow+ (Dentsply Sirona, Konstanz, Germany)	Modified urethane dimethacrylate resin, EBPADMA, TEGDMA, camphorquinone, BHT silanated barium, and strontium glass fillers	Dispense directly in bulk (increments to 4 mm)light-cure for 20 s


Specimens were randomly distributed into two groups according to the setting time of 12 minutes (12 min) or 7 days (7 d) and maintained at 37ºC and 100% humidity during each period. The specimens were randomly distributed into six groups (n = 17):

**SE:** A universal adhesive (Scotchbond Universal Plus, SBU+, Solventum, Maplewood, MN, USA) was applied onto Biodentine in SE mode and light-cured for 10 s (1600 mW/cm^2^, SPEC 3 LED, Coltène/Whaledent, Altstätten, Switzerland).
**ER:** Biodentine was etched with phosphoric acid for 30 s (Scotchbond Universal Etchant, 32%, Solventum), SBU+ applied, and light-cured as above.
**Bur:** Biodentine was roughened with a low-speed water-cooled tungsten carbide round bur (Komet H1 bur ref. 01095K2-027, Komet, Rock Hill, SC, USA) before SBU+ application in SE mode.
**AO:** Biodentine was sandblasted with 27 μm alumina particles (Rondoflex, KaVo Kerr, Biberach, Germany; 2 bar pressure, 10 mm distance). SBU+ was applied, and light-cured for 10 s.
**RMGIC:** A resin-modified glass-ionomer (Vitrebond Plus, Solventum) was placed over Biodentine and light-cured for 20 s.
**SARC:** Biodentine was covered with a self-adhesive resin cement and light-cured for 20 s (RelyX Unicem 2, Solventum).

For groups SE, ER, Bur, and AO, a flowable bulk-fill composite (SDR flow+, Dentsply Sirona, Konstanz, Germany) cylinder was bonded to Biodentine. Both RMGIC and SARC groups were restored in bulk. The restorative material was applied into a cylindrical plastic matrix (2 mm inner diameter, 2 mm height), placed on the Biodentine surface and light-cured (SPEC 3 LED), 1600 mW/cm^2^ for 20s. Subsequently, the plastic tubes were carefully removed with a scalpel. Specimens that debonded during the plastic tube removal were categorized as pretest failures.

### Shear Bond Strength (SBS)

Specimens with interface defects were discarded after evaluation with a stereoscopic microscope at 30× magnification (SZX7, Olympus, Hamburg, Germany). The SBS test was carried out in a universal testing machine (Instron 3345, Instron Mechanical Testing Systems, Norwood, MA, USA; 0.5 mm/min crosshead speed) with a knife-edge plunger targeted at the Biodentine/restorative material interface. SBS was calculated in Mpa, dividing the maximum fracture load (N) by the surface area (mm^2^). Failure mode was assessed by one single operator (30×) and categorized into the following types: adhesive (between Biodentine and restorative material), cohesive (within Biodentine or restorative material), and mixed (combination of adhesive and cohesive modes).

### Micromorphological Analysis and Elemental Mapping

Four blocks were specifically prepared to evaluate micromorphological characteristics of Biodentine surface after adhesive application, acid etching, bur roughening, and AO airborne-particle abrading. An additional untreated block was used as a control. Blocks were observed under a scanning electron microscope (SEM) at different magnifications (50–5000×). Energy-dispersed spectroscopy (EDX) analyses were also performed (Prisma E SEM, ThermoFisher Scientific, Waltham, MA, USA) followed by elemental mapping.

### Statistical Analysis

Pretest failures were included in the statistical analysis as 0 MPa value and equally distributed to both setting times. As data did not follow a normal distribution, Kruskal–Wallis test was performed to detect the influence of the restorative material and strategy on the dependent variable SBS, at both setting times. Partial eta squared (η²H) was calculated as a measure of effect size. According to Cohen’s guidelines (20), η²H values of 0.01, 0.06, and 0.14 indicate small, medium, and large effects, respectively. Posterior comparisons were performed by Mann–Whitney U non-parametric test and Bonferroni correction. Significance level was set at α = 0.05. The percentages of different modes of failure were assessed by a descriptive analysis. Data were analyzed with SPSS version 24.0 (IBM, Armonk, NY, USA).

## RESULTS

### Shear Bond Strength (SBS)

Mean SBS and standard deviation of the experimental groups at both setting times, pretest failures, and mode of failure are shown in Table 2. Pretest failures occurred for all experimental groups when the plastic tubes were removed, except for SE specimens. AO and SARC groups showed 100% pretest failures (0 MPa). At 12-min setting time, RMGIC resulted in 52.9% of pretest failure, whereas ER and Bur groups showed 11.8%. At 7 d setting time, the Bur group showed pretest failures in almost 50% of the specimens.

**Table 2:** Experimental groups mean SBS expressed in MPa (standard deviation, sd)

Groups	SBS (sd)	Pretest failure (%)	Type of failure (%) adh/coh/mix
12 min	7 d	12 min	7 d	12 min	7 d
**SE**	12.1(5.1)^a,A^	8.1(6.2)^a,A^	0	0	6/53/41	12/18/70
**ER**	7.0(5.7)^a,A^	7.7(6.5)^a,A^	11.8	17.6	14/53/33	43/7/50
**Bur**	7.2(5.7)^a,A^	2.3(3.8)^b,B^	11.8	41.1	13/20/67	33/11/56
**AO**	No data	No data	100	No data	No data	No data
**RMGIC**	0.7(1.2)^b,B^	5.7(4.1)^ab,A^	52.9	29.4	25/25/50	16/42/42
**SARC**	No data	No data	100	No data	No data	No data
Percentage (%) of Pretest Failures and Type of Failure: Adhesive (Adh)/Cohesive (Coh)/Mixed (Mix), at 12 minutes (12 min) and 7 days (7d) setting times. Different lowercase letters in columns mean statistically significant differences of SBS values among experimental groups. Different uppercase letters in rows mean statistically significant differences of SBS values between both setting times for each experimental group

Kruskal–Wallis test revealed a significant difference among the experimental groups at 12 min setting time (H (2) = 30.805; p<0.001; η²H = 0.43) and at 7 d (H (2) = 13.497; p<0.001; η²H = 0.16). The RMGIC group achieved the lowest SBS values at 12-min setting time. At 7 d, the SE and ER groups showed the highest SBS, and the Bur group the lowest SBS.

Regarding the influence of the setting time on SBS results, SE and ER groups obtained similar mean SBS. A detrimental effect of time was detected for the Bur group, and a beneficial contribution of delayed restoration was observed for the RMGIC group.

At 12 min setting time, most failures were mixed except for the ER group, in which the cohesive failure of Biodentine was predominant. For 7 d setting time, the SE and Bur groups mainly displayed mixed failures. The ER group showed mixed and adhesive failures equally distributed with less cohesive failures compared with 12 min setting time. The RMGIC group showed the same proportion of cohesive and mixed failures after 7 d setting time.

### Micromorphological Analysis and Elemental Mapping

**Fig 2:**
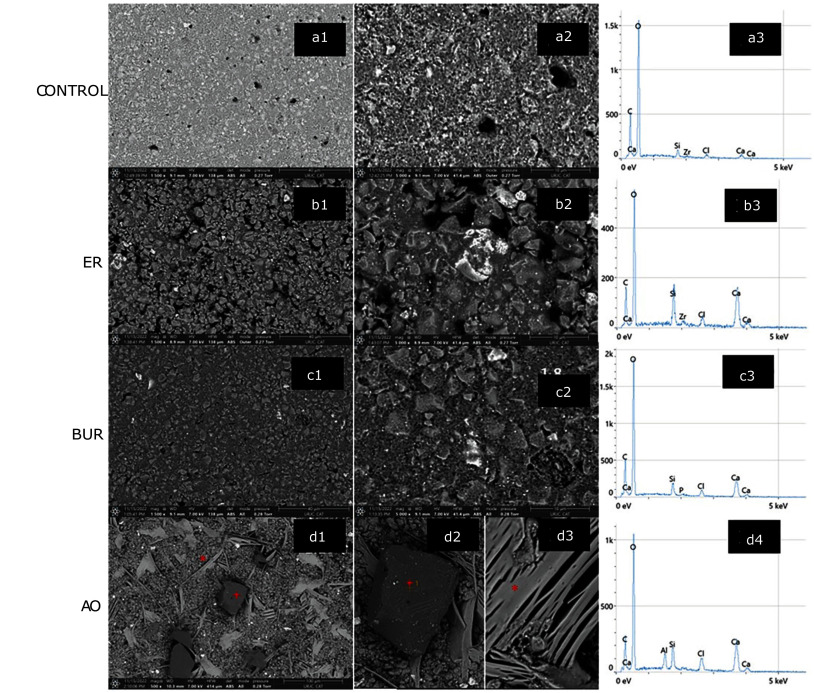
SEM and EDX micrographs of Control, ER, Bur, and AO groups at different magnifications: Control: (a1) SEM 1500×, (a2) SEM 5000×, (a3) EDX; ER: (b1) SEM 1500×, (b2) SEM 5000×, (b3) EDX; Bur: (c1) SEM 1500×, (c2) SEM 5000×, (c3) EDX; AO: (d1) SEM 500× (asterisk: aluminum oxide particle, cross: multiple-layer crystalline particle), (d2) SEM 1500× aluminum oxide particle, (d3) SEM 5000× multiple-layer crystalline particle, (d4) EDX.

SEM micrographs and the corresponding EDX graphics are shown in Figure 2. The control specimen composition (calcium, silicon, oxygen, and carbon) was identified by elemental mapping (Fig 2a.3) and its micromorphology, (Fig 2a.1 to b.2) showed asymmetric particles surrounded by a homogenous area and dispersed porosities, despite being carefully condensed.

**Fig 3:**
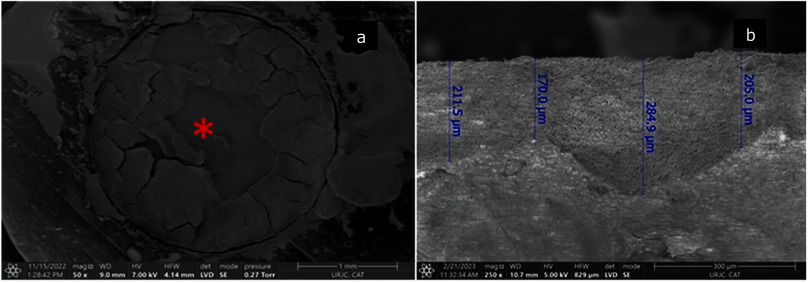
SEM micrograph of acid etching effect on Biodentine (ER group): (a) asterisk (*) indicates surface partially dissolved (50×); (b) acid effect depth (250×).

Etching Biodentine with phosphoric acid (Fig 2b.1 and 2b.2) revealed a heterogeneous surface with an increased number of porosities spread throughout the cement surface compared to the control. Biodentine was partially dissolved by acid, resulting in an irregular surface (Fig 3a) with particles distributed into two strata. Additional micrographs showed that the acid effect reached a depth ranging from 170 to 285 µm (Fig 3b). Regarding the composition, acid etching induced a reduction of oxygen and carbon content (Fig 2b.3).

The morphology obtained with bur roughening (Fig 2c.1 to 2c.2) was similar to that of the ER group. Biodentine was partially removed leading to a non-uniform surface. EDX showed a similar composition to the Control group with elements present in a similar proportion (Fig 2c.3).

**Fig 4:**
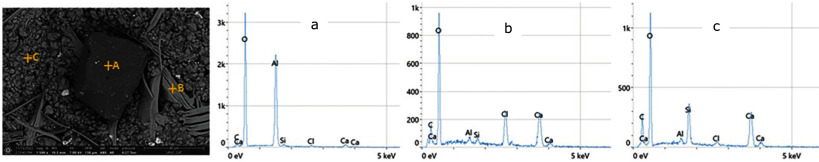
AO group. SEM (1500×) and EDX heterogeneous elemental mapping: (a) Biodentine; (b) aluminum oxide particles (polygonal shape); (c) Multiple-layer particles (calcium and oxygen content)

Elemental mapping of the AO group (Fig 2d.4) identified an increased heterogeneity within Biodentine due to the addition of alumina particles to the surface (Fig 2d.1 and Fig 4). These particles had a polygonal shape (Fig 2d.2), but many others showed a multiple-layer crystalline form (Fig 2d.3), composed of calcium and oxygen (Fig 4c).

## DISCUSSION

The results of the present study corroborate that restorative procedure and setting time significantly influenced bond strength to Biodentine, so both null hypotheses were rejected.

The AO and SARC groups resulted in 100% pretest failures. Airborne-particle abrasion was expected to create an irregular and porous Biodentine surface, increasing mechanical interlocking with the adhesive as well as chemical interaction with 10-Methacryloyloxydecyl dihydrogen phosphate (10-MDP) in the adhesive. Although a drastic surface modification was detected under the SEM, scarce micropores were observed. This airborne-particle abrasion effect was previously reported for MTA restored with composite resin by Samimi et al,^58^ who described fewer pores shallower than those of acid etching, with decreasing bond strength. After airborne-particle abrasion, Biodentine wore easily off resulting in thickness reduction and a heterogenous surface. It displayed the presence of alumina particles and deposition of multilayer crystal particles, probably calcium carbonate crystals eroded by the alumina particles. The main drawback was that all specimens ended up in pretest failures, maybe due to the lack of adhesion of these crystals to Biodentine.

Hursh et al^29^ suggested the use of a dual-cure composite resin as an intermediate material over CSCs to ensure an adequate depth of cure, reporting high bond strengths when Biodentine and ProRoot MTA were tested in comparison with other CSCs. The dual-cure SARC RelyX Unicem 2 was chosen due to excellent previous *in vitro* ^2,34,41^ and clinical results.^55,66^ Its self-adhesive nature allows simultaneous demineralization and infiltration, avoiding surface pretreatment and simplifying the restorative procedure. Nevertheless, RelyX Unicem 2 resulted in 100% pretest failures, being unable to create micromechanical retention on the Biodentine surface or provide chemical bonding

Bur roughening was performed to enhance micromechanical retention of adhesive over Biodentine, resembling alumina airborne-particle abrasion. However, the results showed no advantage in terms of immediate SBS compared with the direct application of an adhesive. After 7 d setting time, the percentage of pretest failures (41.1%) increased.

Clinically, the application of an adhesive on Biodentine can be cumbersome. When rubbed, it fouls and becomes contaminated by CSC debris. Therefore, lining Biodentine with SARC or RMGIC would seal the peripheral dentin and allow proper bonding of composite resin. Nevertheless, these strategies did not improve bonding to Biodentine in the present study. The RMGIC group yielded the lowest SBS values after 12 min setting time, along with a high percentage of pretest failures (52.9%). After 7 d setting time, RMGIC group showed fewer pretest failures (29.4%), although SBS results were not higher in comparison with the application of SBU+. Patel et al^54^ also reported a weak bond strength between Biodentine and glass-ionomer cement, which resulted in lower shear bond strength values and increased interfacial microleakage compared to the Biodentine–composite interface.

In fact, the universal adhesive groups, ER and SE, achieved the highest SBS regardless of previous acid etching and setting time. The effect of phosphoric acid on Biodentine is a matter of interest. Several authors stated that the use of an ER strategy over MTA provides higher SBS than the self-etching mode.^10,67^ This claim has been refuted as phosphoric acid application offers no advantage regarding bond strength, and even a long etching time (over 30 s) could lead to a negative effect on Biodentine properties.^19^ SEM micrographs from the present study showed a pronounced effect of phosphoric acid on Biodentine surface with a material loss ranging from 170 to 285 µm, but its significance was not evaluated.

The universal adhesive used in a SE strategy on a CSC prior to a composite restoration is a simple and effective technique,^50^ but if enamel margins are present, etching is required for a long-term marginal seal and clinical success.^7,64^ The present study found high SBS using SBU+ in SE mode. Chen et al^19^ claimed that adhesives with 10-MDP, such as SBU+, possess chemical affinity for zirconia, resulting in a stronger bond between Biodentine and composite resin. Also, the excellent performance of SBU+ could be attributed to the better wettability of its solvents, enhancing the infiltration into the microporosities of Biodentine.^46,68^ Apart from the 10-MDP content, SBU+ contains the polyalkenoic acid copolymer, known as Vitrebond copolymer (VCP) that can form stable calcium carboxylate salts due to the affinity of its carboxyl groups for calcium ions.^59^ This affinity for the calcium present in Biodentine may contribute to a stable and degradation-resistant adhesive interface.^60^

Each marketed CSC has its own setting time due to ist different composition.^52^ The 12-min time required for Biodentine is still considered clinically long, but shorter than MTA, making it more appropriate for VPT. Previous results on the influence of setting time are contradictory^26,28,53,63^ and a straightforward comparison among materials is not possible. In this study, delayed restorations in the SE and ER groups did not improve SBS. In contrast, Xavier et al^68^ described that a universal adhesive and a self-etching one promoted distinct hybrid layers depending on setting time. More regular adhesive interpenetration was observed in the delayed restorations with higher SBS.

The failure mode reflects bonding effectiveness and, in this study, most of the specimens immediately restored showed either cohesive failures within Biodentine or mixed failures. In contrast, specimens restored after 7 days predominantly exhibited mixed failures, except for those in the ER group. The 7-day ER specimens showed a similar distribution of mixed and adhesive failures. These findings are consistent with previous studies conducted on the adhesive performance of CSCs.^3,4,29,52,53^ Alqahtani et al^4^ stated that bond strength is considered acceptable when failure occurs cohesively within the calcium-silicate-based material, as this indicates a strong adhesion between the CSC and the restorative material. None of the evaluated groups exhibited bond strength values between the CSC and the restorative material that meet the threshold required to counteract the polymerization shrinkage of composite resins.^22^ Consequently, the restoration-dentin interface would remain unaffected, ensuring a peripheral seal around the CSC, which plays a critical role in the success of VPT procedures.

According to our results, restoring Biodentine immediately with SBU+ was the most appropriate procedure, since none of the adhesive strategies tested benefited from longer setting times, in agreement with Odabaş et al^48^ Our *in vitro* results cannot be directly extrapolated to the clinical situation, although they may provide valuable information on the most suitable clinical procedures for VPT. Further laboratory and clinical research testing different restorative materials should be conducted in the future, evaluating their long-term effect on CSC, to enlighten clinicians about the most adequate restorative treatment to cover Biodentine.

## CONCLUSION

Under these study conditions, immediate restoration of Biodentine with a universal adhesive used either in SE or ER mode, followed by bulk-fill composite placement, achieved the highest SBS. RMGIC and SARC did not improve SBS.

### Acknowledgments

The authors express their gratitude to Dr. Jorge Perdigão DMD, MS, PhD, for reviewing and providing advice on the implementation of this scientific article.

#### Expanded Methodology and Reporting

We hereby declare that approval from the Ethical Committee of Rey Juan Carlos University was not required, as the research conducted did not involve living organisms or tissues derived from them.

## REFERENCES
